# Supplementary material for: Hypoxia and hypoxia inducible factor-1α are required for normal endometrial repair during menstruation
Source: Nat Commun. 2018 Jan 23;9:295. doi: 10.1038/s41467-017-02375-6 (PMC5780386; doi:10.1038/s41467-017-02375-6)
Supplement: Supplementary file 1 — Supplementary Information [file 41467_2017_2375_MOESM1_ESM.pdf]

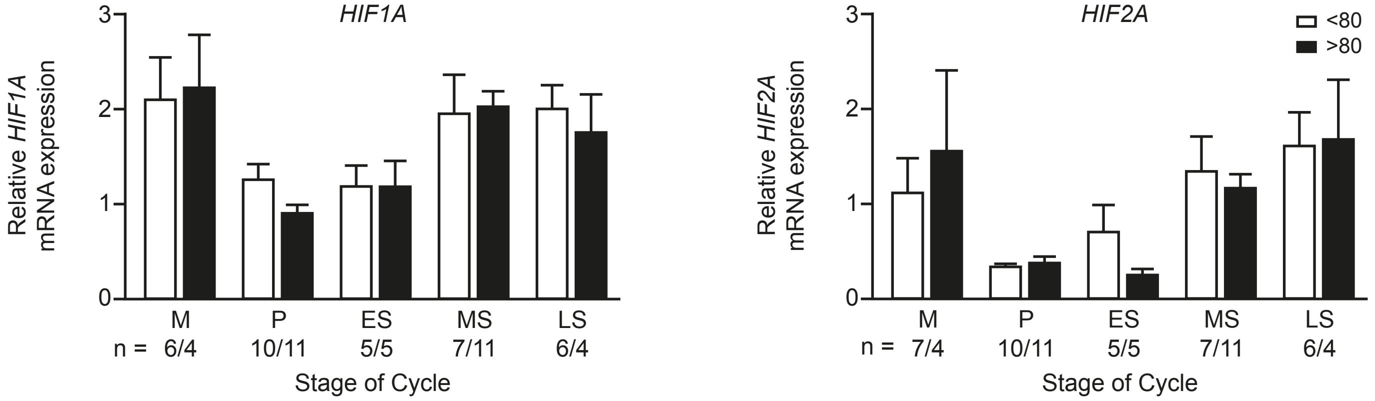

Supplementary Figure 1. *HIF-1α* and *HIF-2α* mRNA concentrations across the menstrual cycle in endometrium from women with normal menstrual bleeding (NMB, <80ml) and heavy menstrual bleeding (HMB, >80ml). M menstrual, P proliferative, ES early secretory, MS mid secretory, LS late secretory. Error bars = SEM.

Score	Morphological Features	Representative H & E
1	Decidualized tissue. Expansion of stromal compartment, presence of decidual cells. Glands pushed towards the myometrium.	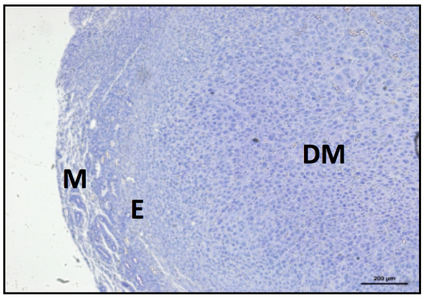
2	Early breakdown. Some loss of structural integrity between decidual cells.<br>Most decidua still intact.	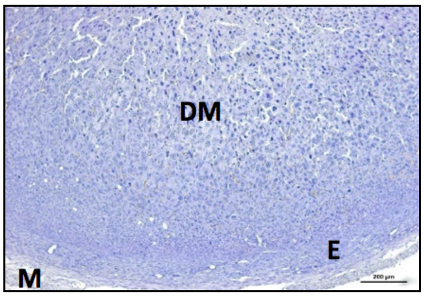
3	Complete breakdown. Complete tissue destruction in decidual zone. No intact decidual tissue. Some sloughing of the endometrium from the myometrium.	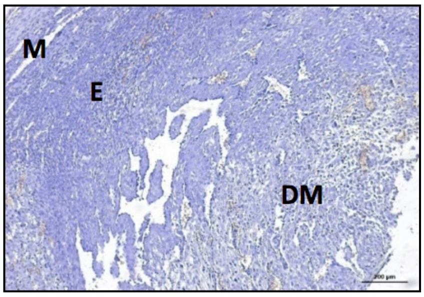
4	Early repair. Beginnings of reepithelialization. Dissociation of necrotic tissue from the myometrium.	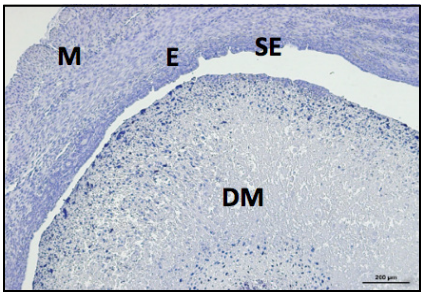
5	Complete repair. Stromal restoration, complete reepithelialization.<br>Small amount of luminal debris.	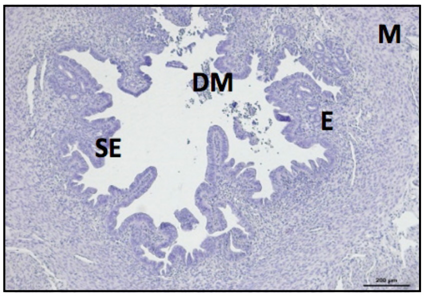

Supplementary Figure 2. Histological breakdown/repair grading system. Histological endometrial breakdown/repair scoring system, adapted from[1] with representative uterine sections displaying histological features of scores 1-5. M myometrium, E endometrium, DM decidualized mass, SE surface epithelium (Scale bar 200um).

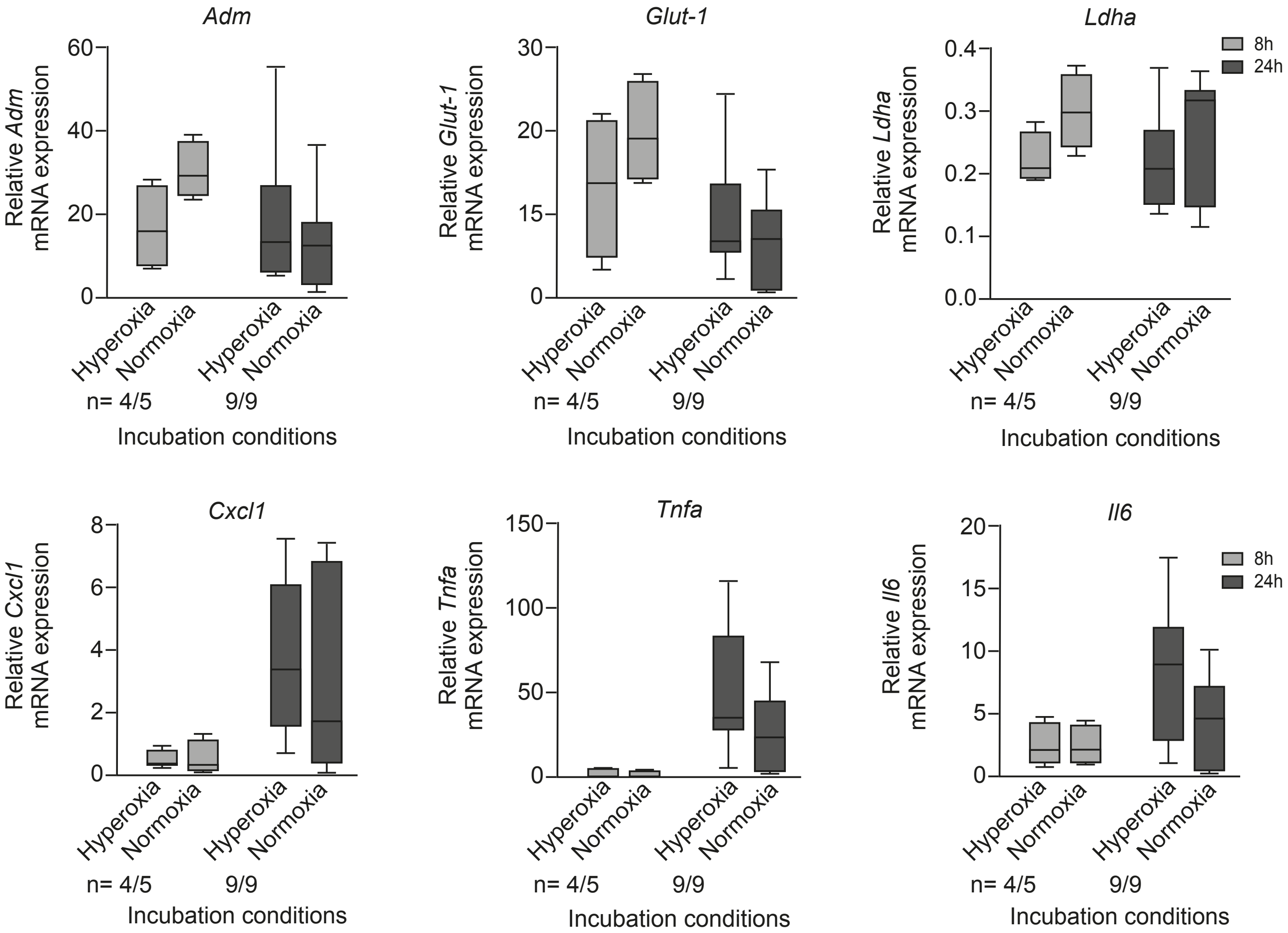

Supplementary Figure 3. *Adm*, *Glut-1*, *Ldha* (HIF regulated) and *Cxcr1*, *Tnfa*, *Il6* (inflammatory mediators) at T8 and T24 in mice with non-hypoxic menstruation (Hyperoxia, 75% O2) and hypoxic menstruation (Normoxia, 21% O2). Box and whisker plots: box represents upper and lower quartiles with horizontal line representing the median, whiskers represent minimum and maximum values.

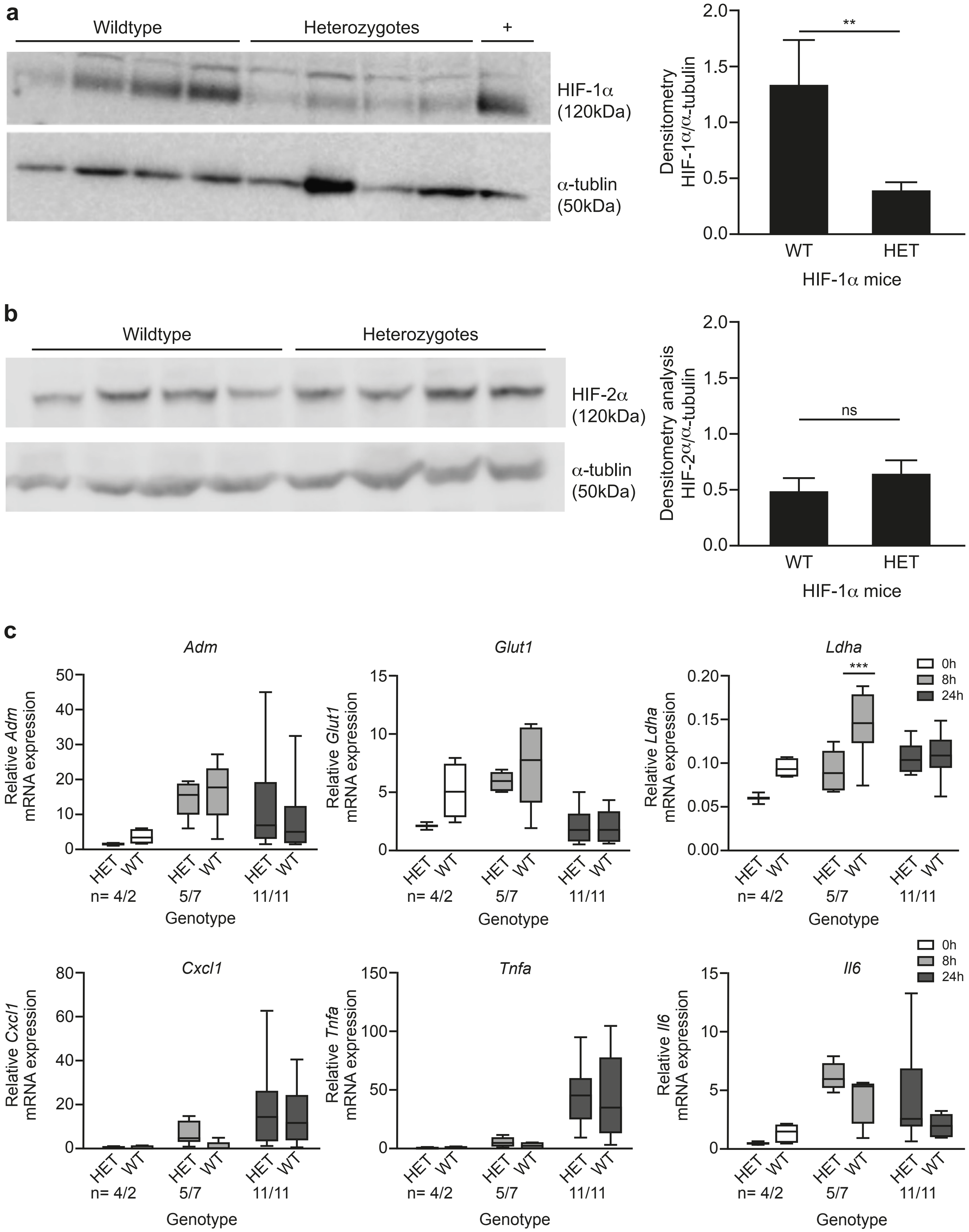

Supplementary Figure 4. (a) HIF-1α protein in endometrial tissue from wild type (WT, HIF-1α+/+) and HIF-1α heterozygous (HET, HIF-1α+/-) mice at T8. + = positive control, protein extract from uterine tissue incubated in hypoxia for 8h. Error bars = SEM. **(b)** HIF-2α protein in endometrial tissue from wild type (WT, HIF-1α+/+) and HIF-1α heterozygous (HET, HIF-1α+/-) mice at T8. Error bars = SEM. **(c)** *Adm, Glut-1, Ldha* (HIF-1 targets) and *Cxcr1, Tnfa, Il6* (inflammatory mediators) at T0, T8 and T24 in HIF-1α heterozygous (HET HIF-1α+/-) and wildtype (WT; HIF-1α+/+) mice. Box and whisker plots: box represents upper and lower quartiles with horizontal line representing the median, whiskers represent minimum and maximum values. **P<0.01, ***P<0.001. (Two-way ANOVA with Sidak's multiple comparisons test).

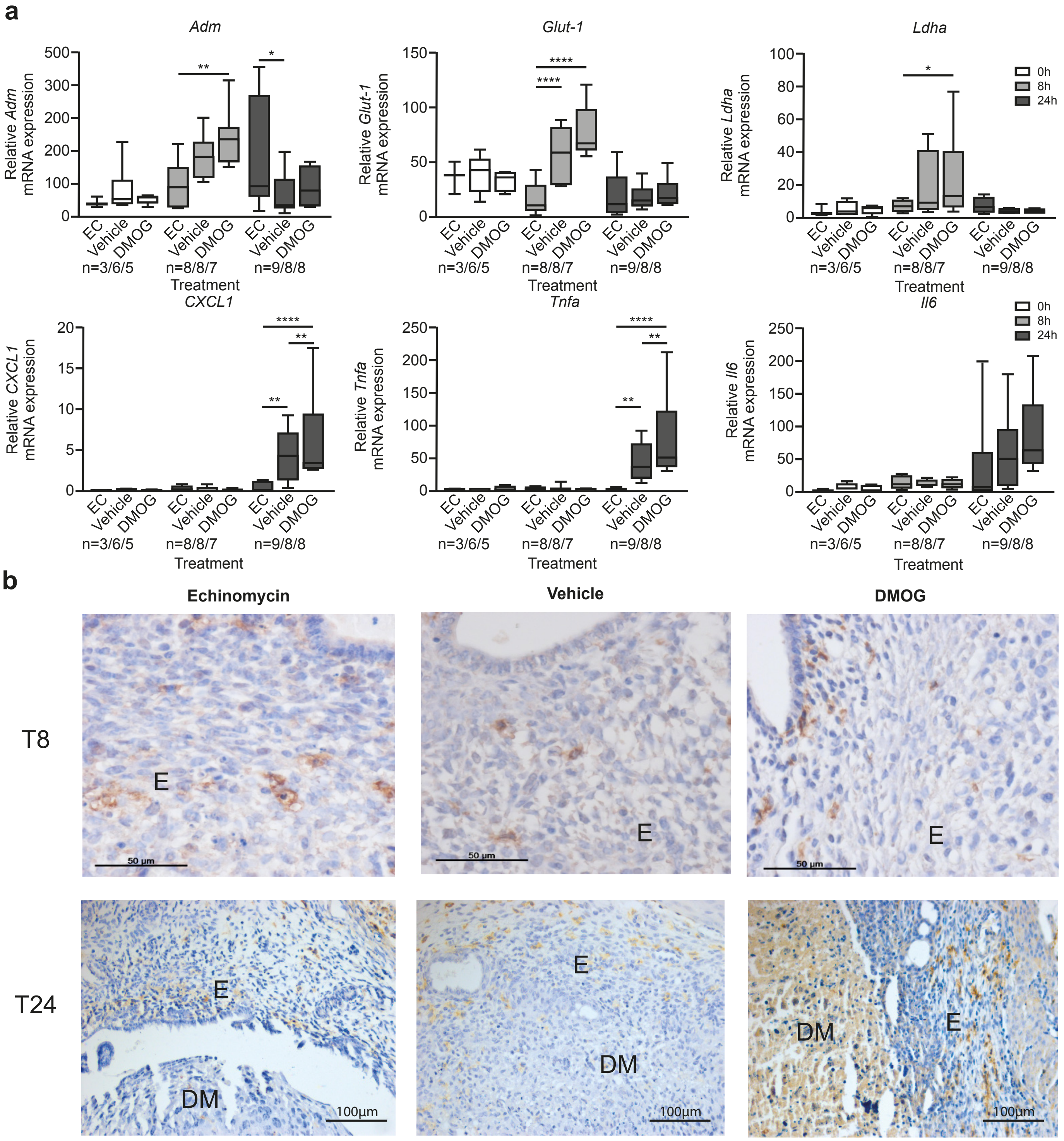

Supplementary Figure 5. (a) *Adm*, *Glut-1*, *Ldha* (HIF-1 targets) and *Cxcr1*, *Tnfa*, *Il6* (inflammatory mediators) at T0, T8 and T24 in mice treated with echinomycin (EC), vehicle or dimethyloxalylglycine (DMOG). Box represents upper and lower quartiles with horizontal line representing the median, whiskers represent minimum and maximum values. *P<0.05, **P<0.01, ****P<0.0001. (Two way ANOVA with Tukey's multiple comparison's test) (**b)** F4/80 staining in endometrium collected 8h (T8) and 24h (T24) following progesterone withdrawal from mice treated with echinomycin (EC: HIF-1 inhibitor), vehicle or dimethyloxalylglycine (DMOG: HIF-1 α stabilizer). E endometrium, DM decidualized mass (Scale bars 50µm upper images and 100µm lower images).

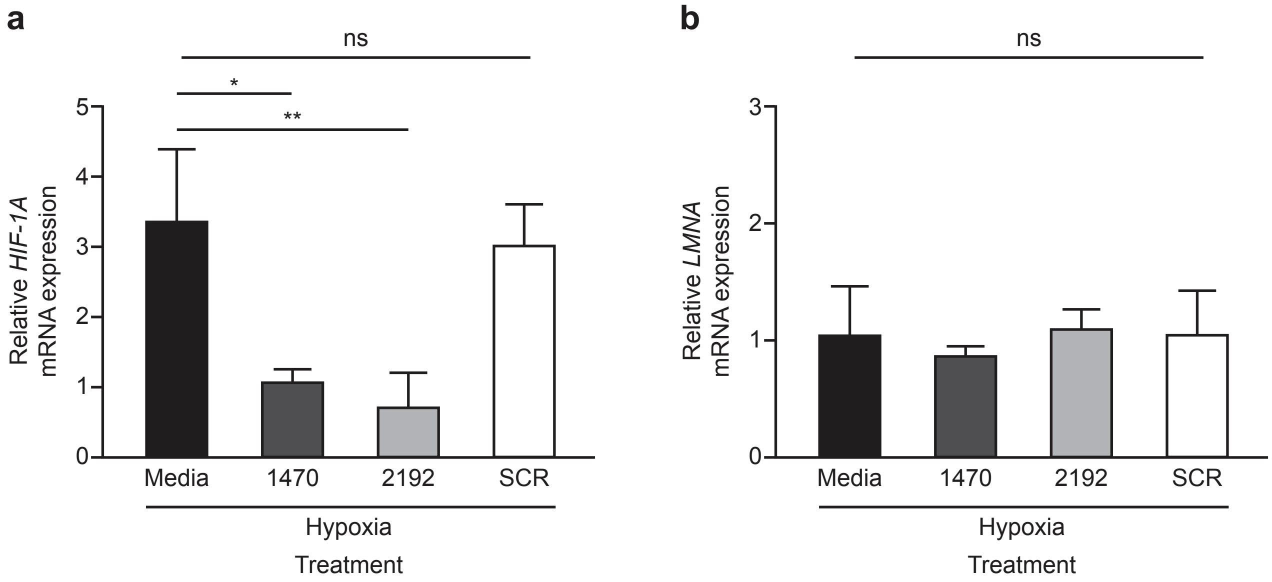

Supplementary Figure 6. Confirmation of HIF-1α silencing in endometrial epithelial cells.
(a) *HIF1A* mRNA in untransfected cells and in cells transfected with a scrambled short hairpin sequence (SCR) and two different short hairpin sequences derived from human *HIF1A* mRNA U22431; bp 1470-1489 (1470) and bp 2192-2211(2192), all incubated in hypoxia (0.5% O2) for 8h. n=3.
(b) *LMNA* mRNA in the same cells to determine sequence specificity of ShRNA constructs.
ns non-significant, *P<0.05, **P<0.01(ANOVA with Tukey's multiple comparison's test). Error bars = SEM.

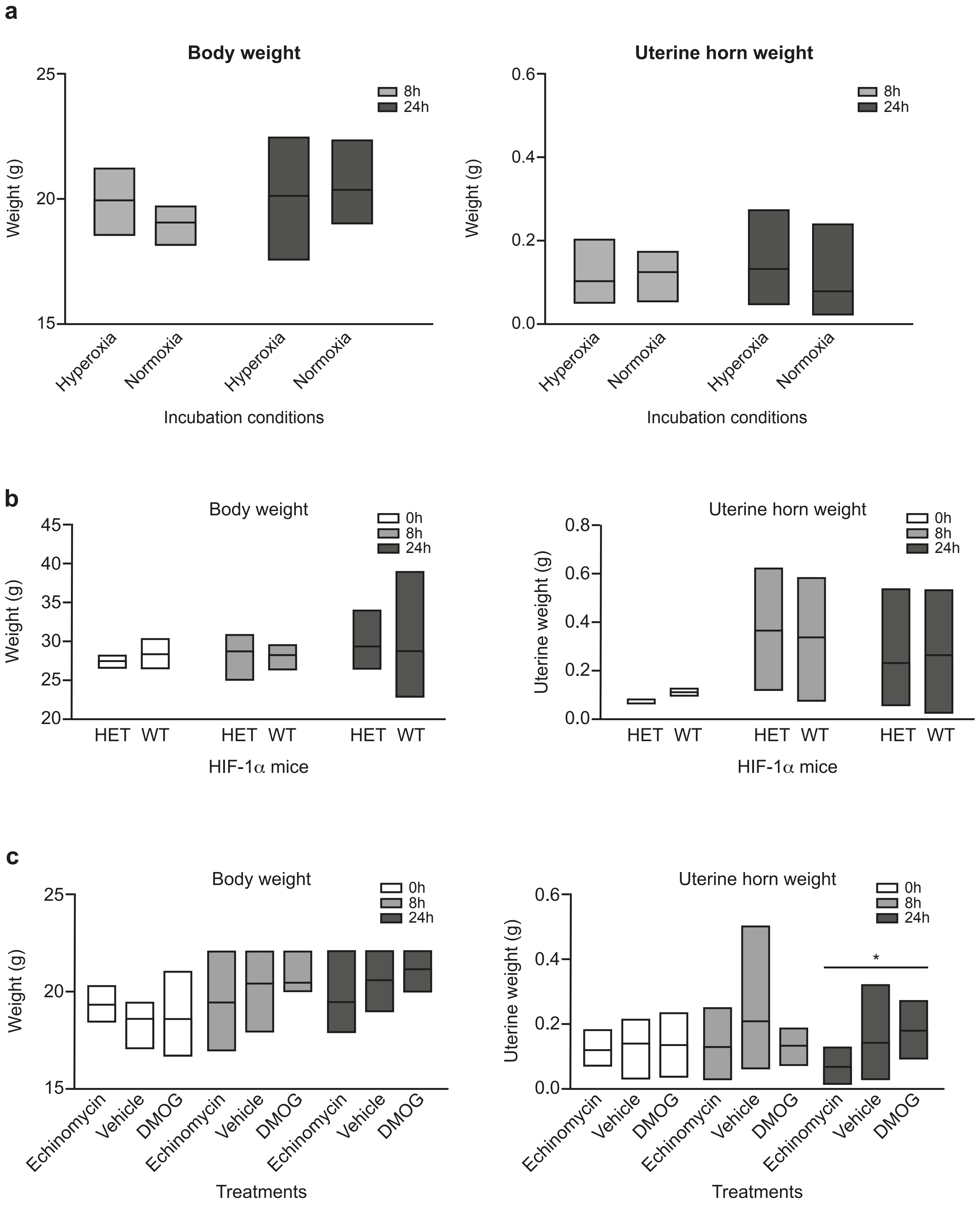

Supplementary Figure 7. (a) Mouse body weights and decidualized uterine horn weights at T8 and T24 in mice incubated in hyperoxia (75% O2) and normoxia (21% O2) at the time of P4 withdrawal. **(b)** HIF-1 α heterozygote (HET HIF-1α$^{+/-}$) and wildtype (WT; HIF-1α$^{+/+}$) mouse body weights and decidualized uterine horn weights at T0, T8 and T24. **(c)** Mouse body weights and decidualized uterine horn weights at T0, T8 and T24 in mice treated with echinomycin (EC), vehicle or dimethyloxalylglycine (DMOG). *P<0.05 (Two-way ANOVA with Tukey's multiple comparison's test). Floating bars represent minimum and maximum weights, line represents mean.

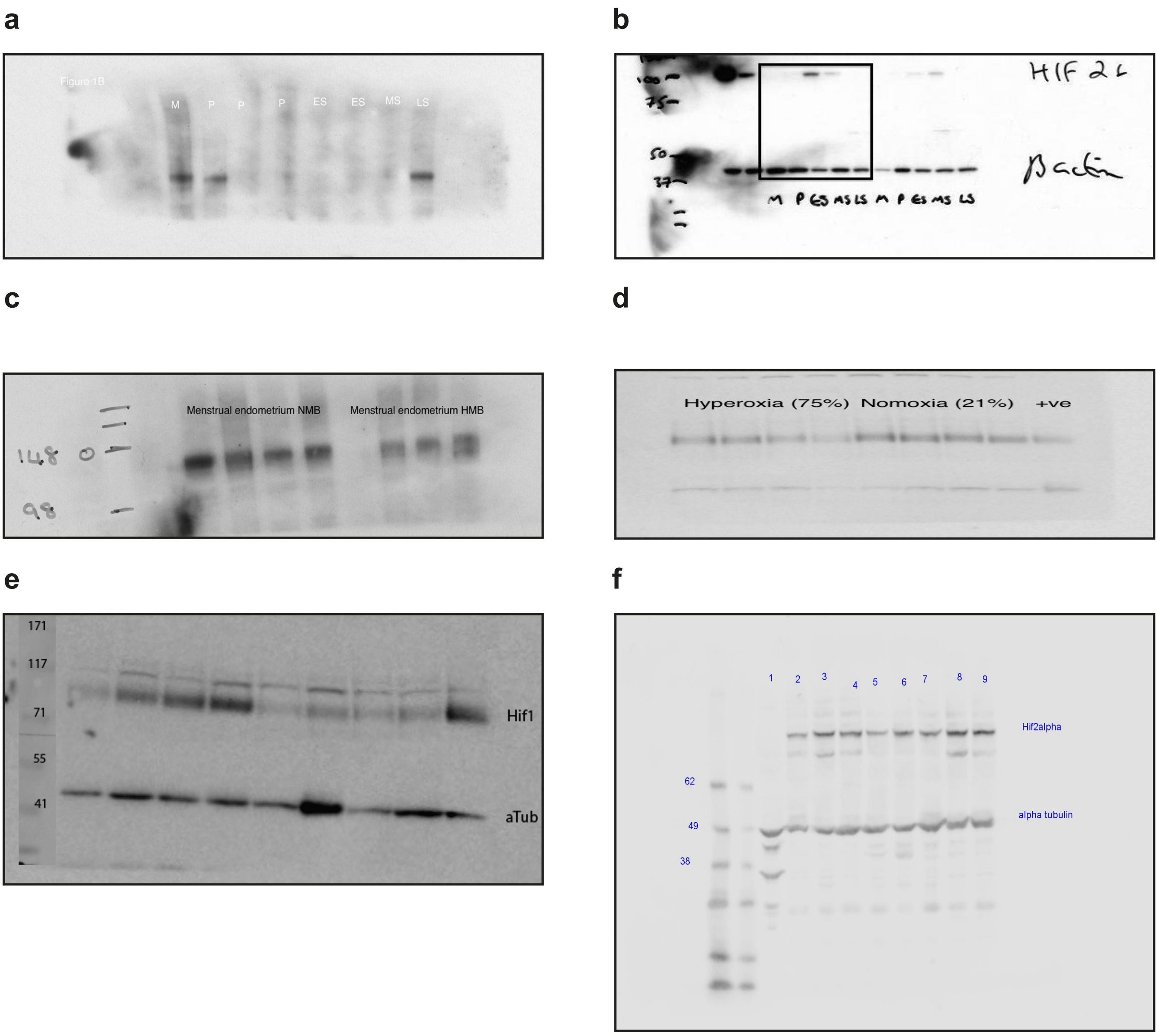

Supplementary Figure 8. Uncropped scans of Western blots. (a) Western blot for HIF-1α in nuclear protein extracts from human endometrial samples taken at different phases of the menstrual cycle (Figure 1b). **(b)** HIF-2α Western blot of endometrial nuclear protein extracts from across the menstrual cycle (Figure 1c). **(c)** HIF-1α Western blot in menstrual phase endometrium from women with NMB and HMB (Figure 1d). **(d)** HIF-1α Western blot of uterine protein extracts at T8 from mice in 75% O2 (hyperoxia = non-hypoxic endometrium) versus 21% O2 (normoxia = hypoxic endometrium) (Figure 3b).
(e) HIF-1α protein in endometrial tissue from wild type and HIF-1α heterozygous mice at T8 (Supplementary Fig. 4a). **(f)** HIF-2α protein in endometrial tissue from wildtype and HIF-1α heterozygous mice at T8 (Supplementary Fig. 4b).

Stage of cycle.	Blood loss (mean, ml)	n-number	Estradiol (mean, pmol/litre)	Progesterone (mean, nmol/litre)
Menstrual	NMB (32)	8	196	3.1
	HMB (184)	7	103	7.4
	No MBL	3	135	6.66
Proliferative	NMB (45)	10	742	4.4
	HMB (246)	11	725	6.0
	No MBL	3	650	4.4
Early secretory	NMB (42)	5	600	31.4
	HMB (137)	5	597	31.0
	No MBL	2	535	47.4
Mid secretory	NMB (34)	8	436	47.1
	HMB (138)	11	486	57.9
	No MBL	1	478	52.7
Late secretory	NMB (39)	6	183	10.9
	HMB (206)	4	224	14.3
	No MBL	1	341	9.2

Supplementary Table 1. Participant serum hormone levels at time of endometrial biopsy and mean menstrual blood loss (MBL). HMB heavy menstrual bleeding (>80ml), NMB normal menstrual bleeding (<80ml).

Gene	Species	Accession number	Forward Primer	Reverse Primer	UPL no.
HIF-1α	Human	ENST00000337138.4	tttttcaagcagtaggaattgga	gtgatgtagtagctgcatgatcg	66
HIF-2α	Human	NM_001430.3	aatcagcttcctgcgaacac	gcttcggactcgttttcaga	55
VEGF	Human	NM_001171622.1	cagcacaacaaatgtgaatgc	ggttcccgaaaccctgag	12
CXCR4	Human	NM_003467.2	ctgtgagcagagggtccag	atgaatgtccacctcgcttt	55
Vegf	Mouse	ENSMUST00000071648.5	ttaaacgaacgtacttgcagatg	agaggtctggttcccgaaa	4
Cxcr4	Mouse	NM_009911.3	tggaaccgatcagtgtgagt	gggcaggaagatcctattga	38
Adm	Mouse	NM_009627.1	ttcgcagttccgaaagaagt	agcgagtcccgtagggtag	42
Glut1	Mouse	NM_011400.3	ggaccctgcacctcattg	gccacgatgctcagatagg	20
Ldha	Mouse	NM_010699.2	ggcactgacgcagacaag	tgatcacctcgtaggcactg	12
Cxcr1	Mouse	NM_008176.3	actccaacacagcaccatga	tggtctgcaggcactgac	49
Tnfα	Mouse	NM_013693.2	ctgtagcccacgtcgtagc	ttgagatccatgccgttg	25
Il6	Mouse	NM_031168.1	gctaccaaactggatataatcagga	ccaggtagctatggtactccagaa	6
Actb	Mouse	ENSMUST00000100497.4	ctaaggccaaccgtgaaaag	accagaggcatacagggaca	64
Rpl13	Mouse	NM_009438.5	tccctgctgctctcaagg	gccccaggtaagcaaactt	41

Supplementary Table 2. PCR Primer details

Supplementary References

Kaitu'u-Lino, T. J., Morison, N. B. & Salamonsen, L. A. Neutrophil depletion retards endometrial repair in a mouse model. *Cell Tissue Res*. **328**, 197-206, doi:10.1007/s00441-006-0358-2 (2007).
